# How good is probabilistic record linkage to reconstruct reproductive histories? Results from the Aberdeen children of the 1950s study

**DOI:** 10.1186/1471-2288-6-15

**Published:** 2006-03-22

**Authors:** Dorothea Nitsch, Susan Morton, Bianca L DeStavola, Heather Clark, David A Leon

**Affiliations:** 1 Department of Epidemiology and Population Health, London School of Hygiene & Tropical Medicine, London, UK; 2 School of Population Health, University of Auckland, Auckland, New Zealand; 3 Dugald Baird Centre, University of Aberdeen, Aberdeen, UK

## Abstract

**Background:**

Probabilistic record linkage is widely used in epidemiology, but studies of its validity are rare. Our aim was to validate its use to identify births to a cohort of women, being drawn from a large cohort of people born in Scotland in the early 1950s.

**Methods:**

The *Children of the 1950s *cohort includes 5868 females born in Aberdeen 1950–56 who were in primary schools in the city in 1962. In 2001 a postal questionnaire was sent to the cohort members resident in the UK requesting information on offspring. Probabilistic record linkage (based on surname, maiden name, initials, date of birth and postcode) was used to link the females in the cohort to birth records held by the Scottish Maternity Record System (SMR 2).

**Results:**

We attempted to mail a total of 5540 women; 3752 (68%) returned a completed questionnaire. Of these 86% reported having had at least one birth. Linkage to SMR 2 was attempted for 5634 women, one or more maternity records were found for 3743. There were 2604 women who reported at least one birth in the questionnaire and who were linked to one or more SMR 2 records. When judged against the questionnaire information, the linkage correctly identified 4930 births and missed 601 others. These mostly occurred outside of Scotland (147) or prior to full coverage by SMR 2 (454). There were 134 births incorrectly linked to SMR 2.

**Conclusion:**

Probabilistic record linkage to routine maternity records applied to population-based cohort, using name, date of birth and place of residence, can have high specificity, and as such may be reliably used in epidemiological research.

## Background

Probabilistic record linkage is increasingly used in health research [1,2] particularly in countries such as Canada and the United Kingdom where there is no system of unique national identity numbers that can be used for linkage purposes [3,4].

Despite a sound theoretical basis for probabilistic record linkage [2], it is often regarded as a second best method in comparison to exact linkage. There is, however, only a limited literature concerned with the reliability and accuracy of probabilistic record linkage [5-9], mainly because there is rarely an independent gold-standard source of information against which the linkage can be judged. Additionally, most assessments of linkage quality have concentrated on the capture of a single event, such as death or cancer registration [5-7]. There have been far fewer evaluations of the more complex situation that arises when attempts are made to link multiple events to the same person, such as births or hospitalisations [8,9].

The aim of this paper is to examine the validity of a probabilistic linkage of women from a Scottish historical cohort born in the early 1950s to records of their own offspring captured by the Scottish Maternity Record system (SMR 2) covering the period 1969–99. This linkage was originally carried out to explore the intergenerational determinants of the birth outcomes among female members of the Aberdeen *Children of the 1950s cohort *[10]. Its validity could be assessed because self-reported information on the offspring of most of the women was also available from a postal questionnaire survey conducted in 2001.

## Methods

### The Children of the 1950s cohort

The *Children of the 1950s *cohort consists of 12150 individuals (5868 females) who were born in Aberdeen between 1950–1956. This cohort was assessed in a cross-sectional survey of childhood cognition conducted in primary schools in 1962 [11].

Their vital status and current area of residence was obtained from the National Health Service Central Registry for 98% of cohort members in 1999. There were 165 deaths for female cohort members. Those women not traced included 139 (2% of females in the cohort) who had emigrated from the UK. The large majority (81%) still resided in Scotland and most (72%) were still in the Grampian region of Scotland that includes Aberdeen.[12]

### Postal questionnaire survey

Sex-specific questionnaires were sent to the study participants starting in May 2001 regardless of place of residence in the UK either by the Scottish Information and Statistics Division (ISD), if resident in Scotland, or by local Health Authorities, if resident in England and Wales. There were 12 women who were not mailed because they were in institutions or dependent on members of the armed forces, and 12 women who were registered with a GP but for whom no precise address information was available to the mailing authorities. In total there were 5540 questionnaires posted to women (detailed information on posting see [13]). The female questionnaires included specific questions on number of births such as: "Have you ever given birth?" and if answered in the affirmative "How many children have you given birth to?". In addition respondents were asked to give details about each birth: "For each child could you please tell us...date of birth, sex, place of birth etc."

### Linkage to Scottish Maternity Record system

The Scottish Morbidity Record (SMR) system was established in 1969. The maternity scheme (SMR 2) contains maternity discharge records that include information on course and outcome of pregnancy as well as socio-demographic data on the mother [14]. A review by ISD found that the completeness of the SMR 2 data in 1969 was only 65%, but increased relatively rapidly in the following years, reaching around 90% in 1976 [15].

The ISD used established probabilistic linkage methods to link the females in our cohort to records in the SMR 2 database. This linkage was based on five fields: surname, maiden name, first and second initial, full date of birth and current postcode (if available to ISD). Of the 5868 women who participated in the *Children of the 1950s *cohort, 5634 women had sufficient information on these fields so that ISD was able to attempt the linkage. The probability matching methodology used has been formally described [16] and is based on the approaches developed by Newcombe and others in Canada [17] and employed by the Oxford Record linkage study [18]. Because of variation and inconsistency in spelling of surnames ISD made use of a mix of two phonetic coding systems that convert phonetically similar surnames to the same linkage key value [16,17]. These were the NYSIIS (New York State Identification and Intelligence System) and Soundex codes (adapted to Scottish surnames).

**Figure 1:**
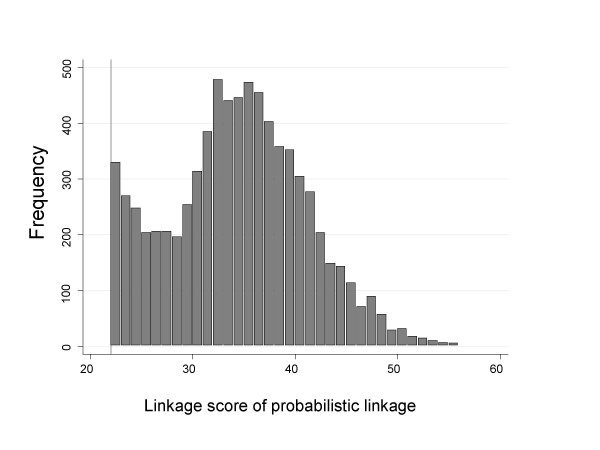
**Frequency distribution of linkage scores greater than 22 used for the probabilistic linkage of females in the *Children of the 1950's* study linkage to SMR 2 maternity records**. The bar width is equivalent to 1.0 unit increase in linkage score. Vertical line: used cut-off score of 22.0.

The linkage process involved the computation of a score for each potential match. The value of this score depended on the probability that each of the five fields agreed. To work this out, weights were computed corresponding to each field. Each weight was defined as the log (to base 2) odds in favour of a match between two records according to that field [2]. Its value depended on how many possible values the field could take, as well as how much misspelling occurred. Records were classified as achieving an acceptable match if the sum of the five weights (the 'linkage score') was greater than a specified value. In this study the threshold value was set at 22.0, this being chosen by ISD. Linkage scores showed a bimodal distribution, with a cluster around values below 22 corresponding to "non-links", another around 38 corresponding to a "definite link" with the "clerical area" defined as the area between the two modes in the distribution. ISD manually examined records in the clerical area in order to minimise the number of false-positive and false-negative links, and determined the cut-off point of 22.0. Exact details on the minimization procedure are not available to us as it involved access to nominal information. Figure 1 shows the distribution of the linkage scores for the records that were accepted as linked (linkage score > 22.0). It is clearly a truncated bimodal distribution, with the cut-point of 22.0 on the right tail of the least likely records.

### Consistency and reliability checks

Consistency checks were performed separately on the information obtained from the questionnaires (n = 5540) and the linked SMR 2 data (n = 5634). Inconsistencies found in the questionnaire data were checked against the original questionnaires and where data entry errors were found these were corrected.

**Figure 2:**
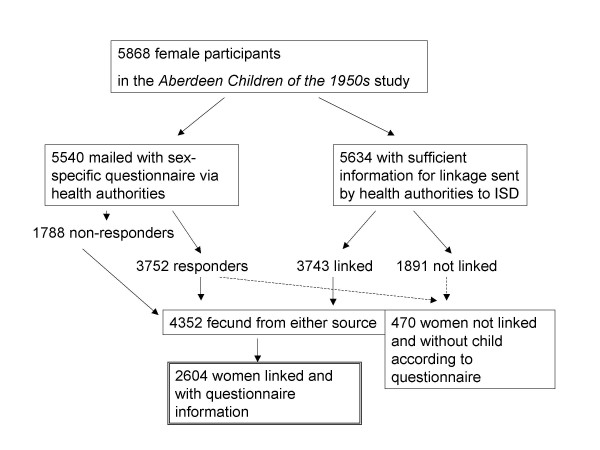
Profile of mailing questionnaires and attempts of linkage with subpopulation considered for further validation of individual births (box with double borders).

The information derived from the two data sources (questionnaire and linkage) was compared in two steps. Firstly, the women were classified on fecundity and according to whether they were present in both, one or neither of the two sources. Secondly the offspring data obtained from mothers who were present in *both *sources were compared and any inconsistencies explored in detail. Therefore the detailed comparison on the number of offspring was conditional on both questionnaire response as well as having been considered for linkage. Figure 2 outlines the dependencies of the validation on the data acquisition processes.

Sensitivity to detect individual offspring data was only calculated for the 5533 offspring born in Scotland to those 2604 women who had information from both sources. This is conditional on questionnaire information and linkage scores (possible only for linked offspring). All analyses were performed using Stata 8 [19].

### Ethical approval

All aspects of the *Children of the 1950's *cohort study, including the questionnaire survey and the linkage to SMR 2, were approved by the Scottish Multi-Centre Research Ethics Committee and other local ethical committees. In addition linkage to SMR 2 was approved by the Scottish Privacy Advisory Committee on the proviso that the linked National Health Service (Scotland) data provided by ISD to our research team were anonymised. As outlined above, we did not have access to personal identifiers from the routine SMR 2 data. The tracing of individuals was done by the health authorities who also mailed the questionnaires for us. ISD worked directly with the local authorities with regards to the linkage procedure. The health authorities then provided us with the anonymised questionnaire information as well as linkage information in one data set stripped of any personal identifiers such as name or day or month of birth of participants.

## Results

### Questionnaire and probabilistic record linkage data

We mailed questionnaires to 5540 women from the cohort of whom 3752 (68%) responded. Response rates were the same for women living in Scotland and those living in England and Wales. Among responders, 3243 reported to have given birth to a total of 7069 infants: 6124 of these babies were stated to have been born in Scotland and 713 in England and Wales. In 33 questionnaires the response to the summary question on total number of offspring did not agree with the detailed information listed by the mothers about each of their children. In the analyses presented below the total number of offspring derived from the detailed information on each child was used in preference to the summary variable.

Linkage to the SMR 2 database was attempted for the 5634 women in the *Children of the 1950s *cohort who had been successfully traced by the National Health Service Central Register. Of these, 3743 (66%) were linked to at least one birth with a linkage score of 22.0 or more. In total there were 7549 linkages to deliveries, all of necessity having occurred in Scotland.

Table 1 shows the distribution of women in the cohort by place of residence in 2003 and social class of their father at the time of their own birth, according to the outcome of the SMR 2 linkage and the postal questionnaire survey. By definition, all those linked to SMR 2 had at least one birth. Of the women who returned a questionnaire 86% reported having had a birth. A larger proportion of linked women (84%) than of questionnaire responders (78%) were still resident in Aberdeen while the reverse held for residence in the UK but outside Scotland (2% versus 11%).

**Table 1:** Frequency and percentages of maternal variables by linkage to the Scottish Maternity Records (SMR 2) and questionnaire response: females in the *Children of the 1950's* Study.

***SOURCE***												
	***SMR 2 (N = 5634) Sent for linkage***				***Questionnaire (n = 5540) Attempted mailings***							

					***Responded***						***No response***	

***Maternal characteristics***	*Linked*		*Not linked*		*No children*		*One or more children*		*Total*			

	*n*	*(%)*	*n*	*(%)*	*n*	*(%)*	*n*	*(%)*	*n*	*(%)*	*n*	*(%)*

**Place of residence in 2003^a^**												
Aberdeen	3147	(84)	1052	(56)	368	(72)	2540	(78)	2908	(78)	1314	(73)
Scotland, not Aberdeen	336	(9)	166	(9)	68	(13)	319	(10)	387	(10)	120	(7)
UK, not Scotland	93	(2)	429	(23)	64	(13)	347	(11)	411	(11)	184	(10)
Emigrated from UK	36	(1)	97	(5)	1	(0.2)	7	(0.2)	8	(0.2)	1	(0)
Other/dead/not traced	131	(3)	147	(8)	8	(2)	30	(1)	38	(1)	169	(9)
												
**Paternal occupation at her own birth^b^**												
												
Non-manual	885	(24)	531	(28)	187	(37)	872	(27)	1,059	(28)	352	(20)
Manual	2579	(69)	1207	(64)	295	(58)	2176	(67)	2,471	(66)	1239	(69)
Other/missing	279	(7)	153	(8)	27	(5)	195	(6)	222	(6)	197	(11)

**Total**	3743	(100)	1891	(100)	509	(100)	3243	(100)	3752	(100)	1788	(100)

^a^Status at National Health Service central Register in 2003

^b^According to occupational status of participant's father at the time of the Reading survey in 1962

Information from both data sources was available for 2604 women. This group was more likely to be resident in Aberdeen and to have had fathers in manual occupations (Table 2). The 609 fecund women who had recorded births in the questionnaire but had not been linked to a SMR 2 record were more likely to have a father with non-manual occupation, and to live outside Scotland [10]. There were a greater number of first births reported before 1971 for these women, compared to other women linked to SMR 2.

**Table 2:** Frequencies and percentages of maternal variables by linkage to the Scottish Maternity Records (SMR 2) and questionnaire response: restricted to women who indicated that they were fecund and for whom linkage to SMR 2 was attempted (excluding deaths before 2000)

***Maternal characteristics***	*Women linked to SMR 2 records and fecund according to questionnaire (N = 2604)*		*Women linked to SMR 2 records only (N = 1139)*		*Fecund women in questionnaire only (no linkage found in SMR 2) (N = 609)*		*Total fecund women from either source (N = 4352)*	
	*n*	*(%)*	*n*	*(%)*	*n*	*(%)*	*n*	*(%)*

**Place of residence in 2003^a^**								
Aberdeen	2249	(86)	898	(79)	297	(49)	3444	(79)
Scotland, not Aberdeen	263	(10)	73	(6)	54	(9)	390	(9)
UK, not Scotland	66	(3)	27	(2)	247	(41)	340	(8)
Emigrated from UK	6	(0.2)	30	(3)	1	(0.2)	37	(1)
Other/dead/not traced	20	(1)	111	(10)	10	(2)	141	(3)
**Paternal occupation of woman at her own birth^b^**								
Non-manual	688	(26)	197	(17)	176	(29)	1061	(24)
Manual	1760	(68)	819	(72)	393	(65)	2972	(68)
Other/missing	156	(6)	123	(11)	40	(7)	319	(7)
**Age at earliest birth **(years)								
<21	705	(27)	316	(28)	220	(36)	1241	(29)
21–25	988	(38)	490	(43)	167	(27)	1645	(38)
26–30	630	(24)	235	(21)	149	(24)	1014	(23)
31–35	194	(7)	65	(6)	43	(7)	302	(7)
36–40	71	(3)	30	(3)	20	(3)	121	(3)
>40	16	(1)	3	(3)	5	(1)	24	(1)
NK	0	(0)	0	(0)	5	(1)	5	(0.1)
**Year of earliest birth**								
<1971	187	(7)	69	(6)	80	(13)	336	(8)
1971–1980	1852	(71)	863	(76)	368	(60)	3083	(71)
1981–1990	512	(20)	185	(16)	143	(24)	840	(19)
1991–2000	53	(2)	22	(2)	18	(3)	93	(2)
NK	0	(0)	0	(0)	0	(0)	0	(0)

^a^Status at National Health Service central Register in 2003

^b^According to occupational status of participant's father at the time of the Reading survey in 1962

### Number of births per woman according to data source

If SMR 2 had complete information on all Scottish births over the entire period when the female cohort members were delivering, and the linkage was 100% accurate and complete, there should be very close agreement between the information given in the questionnaire about births in Scotland and the corresponding maternity records on the reasonable assumption that mothers report details of their offspring reliably and accurately. How far this is the case is investigated in table 3 where the total numbers of births per woman reported in questionnaires and linked to SMR 2 are compared. The main body of table 3 covers the 2604 women for whom information from both sources is available, while the last row refers to the 1139 women with linked SMR 2 data only and the final column to the 1079 women with questionnaire data only (of these, 470 women (44%) did not report a birth).

**Table 3:** Women by number of identified births according to data source (restricted to women for whom SMR 2 linkage was attempted)

		Births per woman according to SMR2 linkage											
Births per woman according to questionnaire		1	2	3	4	5	6	7	8	With linkage to SMR2 and questionnaire		With questionnaire only, no linkage to SMR2	
	**0**	14	5	2	0	0	0	0	0	21	(1)	*470*	*(44)*
	**1**	**397**	9	6	0	0	0	0	0	412	(16)	*139*	*(13)*
	**2**	215	**1210**	24	12	2	0	0	0	1463	(56)	*305*	*(28)*
	**3**	70	94	**374**	12	4	1	0	0	555	(21)	*127*	*(12)*
	**4**	12	15	21	**61**	0	0	0	1	110	(4)	*31*	*(3)*
	**5**	5	2	4	4	**18**	1	1	0	35	(1)	*7*	*(1)*
	**6**	1	0	1	1	1	**1**	0	0	5	(1)	*0*	*(0)*
	**7**	0	1	0	0	0	0	**0**	0	1	(0)	*0*	*(0)*
	**8**	0	0	0	0	1	0	0	**0**	1	(0)	*0*	*(0)*
	**9**	0	0	0	0	0	0	0	1	1	(0)	*0*	*(0)*

With SMR2 linkage and questionnaire	**N (%)**	174 (30)	1136 (47)	432 (18)	90 (4)	26 (0.1)	3 (0)	1 (0)	2 (0)	2604 (100)	(100)	*1079*	*(100)*

With SMR2 linkage, no questionnaire	**N (%)**	*338 (30)*	*513 (45)*	*213 (19)*	*52 (5)*	*15 (0.1)*	*6 (0)*	*2 (0)*	*0 (0)*	*1139 (100)*			

Among the fecund women with data from both sources, 79% (2061/2604) had the same total number of births per woman reported by questionnaire and SMR 2. These women, identified in bold on the diagonal of Table 2, delivered a total of 4279 births. For 94 women, listed above the diagonal, there were 135 more births linked than reported in the questionnaire. On the other side, listed below the diagonal in table 2, for 449 women there were 594 fewer linked births than stated in the questionnaire.

In summary, for 79% of the 2604 women with information from both sources there is agreement on total number of births per woman. For 4% there were more linked births than stated in the questionnaire, and for 17% there were more births reported in the questionnaire than linked to SMR 2.

### Matching of individual births across data sources

Even when the total number of births from questionnaire and SMR 2 were in agreement, there were still some differences in the sex and date of birth of the infants as reported in the two sources. This is shown in table 4 with the number of births split up according to whether there was an exact match on mother, sex and date of birth in both data sources (table 4, first column).

**Table 4:** Numbers of births^a ^(and corresponding median linkage scores) by whether or not there was a corresponding match between details (mother, sex, year of birth) in SMR 2 and questionnaire, classified according to differences in total number of births/mother between both sources.

		***Total number of births to mother***			
		
**Source of individual birth details**		**Same in SMR 2 and questionnaire**	**More in SMR 2**	**More in questionnaire**	**Total Number of births**
**In SMR 2 and questionnaire (exact match)**	Number of births	4166	160	604	4930
	*Median linkage score (25^th^, 75^th ^centile)*	*35 (31, 40)*	*35 (32, 40)*	*32 (26, 36)*	
**In SMR 2 only (exact match not found in questionnaire)**	Number of births	113	131^b^	39	283
	*Median linkage score (25^th^, 75^th ^centile)*	*36 (32, 41)*	*24 (23, 30)*	*27 (24, 41)*	
**In questionnaire only (exact match not found in SMR 2)**	Number of births	113	4	633	750
	*Median linkage score (25^th^, 75^th ^centile)*	*n.a.*	*n.a.*	*n.a.*	

	**Total number of mothers**	2061	94	449	2604

^a ^restricted to data on mothers for whom information was available from both linkage to SMR 2 and questionnaires.

^b^Out of 135 additional births found above the diagonal in table 3, 4 births were in fact matches according to the overall information given by the mother in the questionnaire (they specified to have children, but omitted detailed information)

n.a.: not applicable

The top cell of this column shows that in the two data sources there were 4166 exact matches. For 113 births there was a birth not matching to the information recorded either in questionnaire or in SMR 2 (the two next cells below). On closer examination most of these mismatches appear to be data entry errors, either from the questionnaire or from the maternity records: for 73 infants year of birth differed but day and month of birth agreed, for 22 there were sex mismatches, and for 18 missing entries either for sex or year of birth. The likelihood of these being data entry errors is supported by the distribution of their linkage scores (table 4, in italic below the number of births): the median value for these 113 births was very similar to those of the 4166 births that were consistently identified by questionnaire and SMR 2 and were also well above the threshold linkage score of 22.0.

The 94 women for whom more births were linked than reported in the questionnaire (table 4, second column), had 131 births from SMR 2 that did not match with those stated in the questionnaire. Apart from 4 mismatches where the mothers had stated totals that were different from the detailed information given that were possibly due to entry errors, most (127 births) were likely to be "false positive" linkages, as reflected by their median linkage score of 24.

The 449 women for whom fewer births were linked than reported in the questionnaire (table 4, third column), had 604 matched births identified by both questionnaire and linkage, with a high median linkage score. A further 39 births had been linked to them but did not correspond to any of the births stated in the questionnaire and these tended to have a low median linkage score. A detailed investigation showed that data entry errors in year of birth or sex could potentially explain 32 of these mismatches. The remaining 7 births were implausible, and are likely to represent false positive linkages. Thus, assuming that there were 32 data entry errors, 601 out of 633 births were stated only in the questionnaire. About a quarter of these 601 were reported in the questionnaire to have been delivered outside Scotland (147/601). As the SMR 2 system only covers Scottish deliveries these excess births are therefore explicable. Of the remaining 454 births, 442 were born in Scotland before 1976 which is prior to SMR 2 having established complete national coverage.

In summary, information from SMR 2 and as provided by the mothers in the questionnaire was identical for 89% of births occurred in Scotland (4930/5533). There were 3% births (149/5533) with possible errors in the questionnaire (see above: 131 + 4 + 32 = 149). Hence, the linkage had overall a sensitivity of 92% for detecting all the births delivered in Scotland, and led to 134 assigned birth records which were inconsistent with the information provided by the mothers.

### Effects of shifting the cut-off for linkage

Treating the information obtained from the questionnaires as correct, the choice of a linkage cut-point at 22.0 led to 134 incorrectly assigned births ('false positives') and to 454 missed births in Scotland ('false negatives'). Higher cut-off points would have led to smaller numbers of false positive linkages but larger numbers of false negatives (Table 5). For example increasing the value of the cut-off point used to define a successful linkage from 22.0 to 24.0 would reduce the number of false positive cases by 50%. The sensitivity corresponding to the different cut-points can be also be computed by comparing the numbers of false negatives to the number of questionnaires that reported Scottish births. So changing the cut-point to 24.0 would have reduced the sensitivity from 92% to 86%. Since the 2604 women identified in both data sources have by definition at least one birth, it is impossible to calculate the equivalent specificity values.

**Table 5:** False positive links, missed birth records and sensitivity of probabilistic record linkage by linkage cut cut-off values; females in the *Children of the 1950's* study linkage to SMR 2 maternity records.

***Linkage cut-off point***	***Number of false positive links^a^***	***Number of missed birth records^a^***	***Sensitivity^a^***
**22**	134	454	92%
**23**	91	617	89%
**24**	68	775	86%
**25**	61	926	83%
**27**	37	1169	84%
**29**	32	1422	74%
**31**	28	1777	68%
**35**	10	2940	53%
40	3	4383	21%

^a ^Calculated only for those 2604 women where information from both sources is available and who gave birth to 5533 children in Scotland.

## Discussion

We have examined the results of a probabilistic linkage between a research database holding single individual records and an administrative database potentially holding multiple corresponding records. The first database contained information on the females of the *Children of the 1950's *study, the second, SMR 2, details of most infants born in Scotland since 1969. Overall we found that the probabilistic record linkage yielded highly comparable linked births to those reported by the cohort members who were re-contacted by postal questionnaire in 2001. When focussing on fecund women and treating the questionnaire information as the "gold standard", the sensitivity to detect all individual births was 92% while 134 births were erroneously linked i.e. were false positives.

The quality of linked data depends on the quality of the data collection of the original datasets. We found that there was a gap between the overall percentage of fecund women between the questionnaire (86%) and SMR 2 (66%). Since SMR 2 started in 1969 and is deemed not to have reached national coverage before 1976, our finding that most of the births missed by the linkage were born before 1976 is not surprising. Other missed links were births that occurred outside Scotland: thus the process has limitations when it involves a mobile population that cannot be followed up by the administrative database. Our findings are consistent with the observation of the higher likelihood of migration out of Scotland for the more advantaged social groups [10,20].

Our comparisons of the information held in the linked and questionnaire data may be affected by bias because not all traced women returned a questionnaire (the response rate was 68%). The problem of questionnaire non-response led to some difficulties in computing a specificity for linkage. Furthermore, responders and non-responders in our study had different features [13]. However, recall bias is unlikely to affect the questionnaire data that concern birth histories [21,22] and thus it seemed sensible to treat that information as the reference for assessing the reliability of the linkage. Using sensitivity analyses, we examined how both migration might have affected the validity of linkage as a proxy for fecundity, as well as to which extent questionnaire non-response might have led to wrong estimates of sensitivity of linkage. We came to the conclusion that the linked data seemed to be relatively robust [23].

Our results are comparable to those found in other studies which had similar datasets for comparison. The follow-up of patients in the WOSCOPS trial was performed at the same time as a probabilistic record linkage to records of subsequent hospital admissions in Scotland-a study that also involved linkages carried out by ISD [8]. The probabilistic record linkage was very successful, with only 52 events related to 24 subjects who were wrongly linked and 95% of hospital admissions correctly identified by the record linkage system. The slightly better results might be due to the fact that this study had a more stable population and that linkage was related to events at a time when the data base used for linkage had almost complete recording. In a study conducted in Brazil, data from a household survey of on hospital care usage were compared to probabilistic linkage to hospital records [9]. The investigators found only 46% agreement between hospital records and survey information. However this low estimate of agreement may not reflect the quality of record linkage per se, because there were massive deficiencies in the hospital recording system (e.g. emergency admissions were not recorded).

Overall a universal identifier – such as a personal health insurance number-linking all administrative and research databases would seem preferable. Such a single identifier is the norm in all Scandinavian countries and is generally used for deterministic linkage, i.e. linkage restricted to 100% agreement. The conditions for its success are nevertheless strongly dependent on the quality of the coding of the personal identifier and its availability. When such a system is not available, probabilistic linkage based on several fields (including accounting for possible misspellings) provides a satisfactory substitute [22,23]. There are methods to calculate the predictive value associated with probabilistic linkage with respect to the probability that the linked event will occur, but these methods apply to single, non-recurrent events [24]. Where there is a number of possible links per person, as for the number of births of a woman, these methods are not applicable. We have shown that probabilistic linkage led to the identification of a high proportion of Scottish births for the women in our cohort. We have also demonstrated that shifting the linkage thresholds decreased the possibility of false positive results, but had an associated loss of sensitivity. In this study, we used the maximum set of identifying matching variables common to both SMR 2 and female participants of the *Children of 1950*s study. Of course, the hypothetical inclusion of more matching variables (unavailable to us in practice) could have resulted in a higher sensitivity with high specificity at a higher linkage threshold.

In the context of epidemiological research the advantages and disadvantages of follow-up using routine record linkage need to be compared with active follow-up methods, such as questionnaires, or regular invitations to health checks. The associated costs and the amount and quality of information to be gained from these alternative approaches must be weighed against each other. Overall, especially when there is a long lag between the original study recruitment and the follow-up, record linkage can be useful if the quality of the linked dataset is acceptable. Despite an ever increasing trend to the recording of national unique numerical identifiers we would argue that probabilistic record linkage using traditional person identifiable data still provides the flexibility to link with data sources not compatible with such a key. Perhaps a linkage system that best serves the demands of epidemiological research in the future could readily incorporate a combination of exact matching on universal identifier corroborated by probability matching techniques on the traditional identifiers.

## Conclusion

In conclusion this example of probabilistic record linkage between individual research data and a database of maternity discharge records produced data on births to cohort members that was of good validity. In particular, it identified few false positives. Those births that were missed were largely explicable in terms of the known limitations of the routine database.

## Competing interests

The author(s) declare that they have no competing interests.

## Authors' contributions

D Nitsch performed all analyses, wrote the first draft and produced the final version of the paper. S Morton produced the intergenerational database for the *Children of the 1950s *cohort. All authors contributed to the development of ideas for the paper and to its drafting.

## Pre-publication history

The pre-publication history for this paper can be accessed here:


